# Comparison of Burrowing and Stimuli-Evoked Pain Behaviors as End-Points in Rat Models of Inflammatory Pain and Peripheral Neuropathic Pain

**DOI:** 10.3389/fnbeh.2016.00088

**Published:** 2016-05-10

**Authors:** Arjun Muralidharan, Andy Kuo, Meera Jacob, Jacintha S. Lourdesamy, Lara Melo Soares Pinho De Carvalho, Janet R. Nicholson, Laura Corradini, Maree T. Smith

**Affiliations:** ^1^Centre for Integrated Preclinical Drug Development, The University of QueenslandBrisbane, QLD, Australia; ^2^Department of CNS Diseases Research, Boehringer Ingelheim Pharma GmbH & Co. KGBiberach, Germany; ^3^School of Pharmacy, The University of Queensland, St Lucia CampusBrisbane, QLD, Australia

**Keywords:** burrowing, CCI, FCA, inflammatory pain, mechanical allodynia, mechanical hyperalgesia, paw volume, peripheral neuropathic pain

## Abstract

Establishment and validation of ethologically-relevant, non-evoked behavioral end-points as surrogate measures of spontaneous pain in rodent pain models has been proposed as a means to improve preclinical to clinical research translation in the pain field. Here, we compared the utility of burrowing behavior with hypersensitivity to applied mechanical stimuli for pain assessment in rat models of chronic inflammatory and peripheral neuropathic pain. Briefly, groups of male Sprague-Dawley rats were habituated to the burrowing environment and trained over a 5-day period. Rats that burrowed ≤ 450 g of gravel on any 2 days of the individual training phase were excluded from the study. The remaining rats received either a unilateral intraplantar injection of Freund's complete adjuvant (FCA) or saline, or underwent unilateral chronic constriction injury (CCI) of the sciatic nerve- or sham-surgery. Baseline burrowing behavior and evoked pain behaviors were assessed prior to model induction, and twice-weekly until study completion on day 14. For FCA- and CCI-rats, but not the corresponding groups of sham-rats, evoked mechanical hypersensitivity developed in a temporal manner in the ipsilateral hindpaws. Although burrowing behavior also decreased in a temporal manner for both FCA-and CCI- rats, there was considerable inter-animal variability. By contrast, mechanical hyperalgesia and mechanical allodynia in the ipsilateral hindpaws of FCA- and CCI-rats respectively, exhibited minimal inter-animal variability. Our data collectively show that burrowing behavior is altered in rodent models of chronic inflammatory pain and peripheral neuropathic pain. However, large group sizes are needed to ensure studies are adequately powered due to considerable inter-animal variability.

## Introduction

Chronic pain, that affects ~15–20% of the adult population globally (van Hecke et al., 2013), is underpinned by complex cellular and molecular pathophysiological mechanisms (Basbaum et al., 2009). Poorly relieved chronic pain not only affects the quality of life of patients and their care-givers, it also imposes a significant socioeconomic cost (Woolf, 2010).

Rodent models of individual chronic pain conditions are crucial to improving our collective understanding of the specific pathobiological mechanisms and for screening new molecules as potential analgesic or adjuvant agents (Mogil et al., 2010). Over the past two decades, numerous novel ‘pain targets’ including receptors, ion-channels and enzymes have been identified and implicated in the pathobiology of chronic pain. However, most compounds that modulate these targets failed to show analgesic efficacy in proof-of-concept human clinical trials, despite promising preclinical data (Smith and Muralidharan, 2015). This perceived failure of drug candidates in clinical trials, has led to calls for the replacement of rodent pain models with studies in human volunteers (Langley et al., 2008).

Pain, a subjective phenomenon, is inferred based upon behavioral responses in rodents and self-reported pain severity ratings, that encompasses intensity of the nociceptive stimulus and its resultant affective/emotional response, in humans (Muralidharan and Smith, 2011; Tappe-Theodor and Kuner, 2014). In the preclinical setting, multiple reflex-withdrawal based behaviors have been established as pain behavioral end-points in rodents (Percie du Sert and Rice, 2014). However, the validity of solely using stimuli-evoked methods for assessing pain behaviors in rodents has been questioned critically regarding their ability to mimic spontaneous ongoing pain, numbness and dysesthesia reported by many patients with various chronic pain states (Maier et al., 2010; Bennett, 2012; Percie du Sert and Rice, 2014; Tappe-Theodor and Kuner, 2014). Hence, ethologically-relevant rodent behaviors such as burrowing, that are altered by pain and reinstated by analgesics, have been proposed as a potential means to mimic spontaneous pain in humans (Percie du Sert and Rice, 2014).

Rats and mice *(Mus musculus)*, the most commonly used laboratory species for experimental pain models, are well-known burrowers as this behavior is innate and highly conserved due to its importance in defense against predators (Deacon, 2006). Burrowing behavior is regarded as a measure of “global well-being” in rodents since it is affected by a range of diverse perturbations such as brain lesions (Jirkof, 2014), inflammation (Jirkof et al., 2013), and activation of the immune system (Teeling et al., 2007). A simple experimental setup for assessing burrowing behavior of rats or mice has been described (Deacon, 2006). In this experiment, a rodent moves a substrate (e.g., gravel or sand) out of a container via coordinated hind and fore-limb movements, and the amount of substrate displaced is measured (Deacon, 2006). Previous work by others has shown this behavior to be altered by various pain states and reinstated by clinically proven analgesics, thereby confirming the predictive validity of this assay (Jirkof et al., 2010; Andrews et al., 2012; Lau et al., 2013; Rutten et al., 2014a,b). Importantly, it is also suggested that burrowing measures spontaneous ongoing pain, rather than evoked pain, as the amount of substrate burrowed was not correlated with evoked paw withdrawal measures (Andrews et al., 2012). Since chronic pain can have a profound impact on a patient's well-being, measuring the effect of chronic pain in rodents on burrowing behavior that is thought to be an indicator of spontaneous ongoing pain as well as well-being in these animals, may offer a significant advantage regarding assessment of the global impact of pain in the preclinical setting (Andrews et al., 2011). However, it is also important to carefully assess the validity of this innate behavioral assay between laboratories located in different countries around the world before considering it as a replacement for reflex-based limb/tail withdrawal assays in response to applied stimuli, or as a surrogate measure of pain.

Hence, the aim of our present investigation was to compare the utility of burrowing behavior relative to that of mechanical stimuli-evoked behavioral pain measures, in rat models of Freund's complete adjuvant (FCA)-induced inflammatory pain and chronic constriction injury (CCI) of the sciatic nerve induced peripheral neuropathic pain.

## Materials and methods

### Animals

This study was conducted in accordance with the guidelines set out in the Australian Code of Practice for the Care and Use of Animals for Scientific Purposes (NHMRC, 2013). Animal ethics approval was obtained from the Animal Ethics Committee of The University of Queensland for the studies described herein and our experiments adhered to the guidelines of the Committee for Research and Ethical Issues of the International Association for the Study of Pain.

Groups of male Sprague-Dawley (SD) (180–200 g) rats were purchased from the Animal Resources Centre (Perth, WA, Australia). Upon arrival at our facility, rats were housed in groups of two to three in a temperature-controlled room (21°C±2°C) with a 12 h/12 h light-dark cycle. Environmental enrichment comprised placement of rodent hutches and rat chew sticks in all home cages. Standard rodent chow and water were available *ad libitum*. Rats were acclimatized for at least 3 days prior to initiation of any experiments.

### Induction of inflammatory pain

Inflammatory pain was induced in rats by unilateral intraplantar (i.pl.) injection of FCA, as previously described (Edwards et al., 2007). Briefly, whilst anesthetized with 3% isoflurane delivered in oxygen, SD rats (200–250 g) received an i.pl. injection of 150 μl of FCA (Sigma Aldrich, MO, USA) into their left hindpaws. The corresponding groups of sham-rats received 150 μl i.pl. injections of saline. Following i.pl. injection, rats were returned to their home cages and were monitored for general health and body weight changes twice-weekly until study completion at day 14 post-FCA or saline injection.

### Induction of peripheral neuropathic pain

Chronic constriction injury of the sciatic nerve was used to induce peripheral neuropathy in rats, according to a published method (Bennett and Xie, 1988). Briefly, male SD rats (200–250 g) were anesthetized with 3% isoflurane delivered in oxygen. After shaving the left thigh, the skin was cleaned using 70% ethanol. Next, a small incision was made through the biceps femoris to expose the sciatic nerve. Subsequently, four loose ligatures (~1 mm apart) were tied proximal to the trifurcation of the sciatic nerve using silk sutures. The muscle and skin were closed, and the animals were monitored closely during surgical recovery. For rats that underwent sham surgery, the sciatic nerve was exposed but not ligated. Following surgery, the rats were returned to their home cages and were monitored for general health and body weight changes twice weekly until study completion at day 14 post-CCI or -sham surgery.

### Behavioral studies

All behavioral experiments described herein, except burrowing, were conducted between 09:00 and 14:00 h. Burrowing experiments were carried between 15:30 and 17:00 h (12 h light (06:00–18:00 h)/dark cycle (18:00–06:00 h).

#### Assessment of hindpaw volumes

For rats administered an i.pl. injection of either FCA or saline, ipsilateral (injected side) and contralateral (non-injected side) hindpaw volumes (PV) were measured using a Plethysmometer (Ugo Basile, Italy). Measurements were done just prior to FCA or saline injection (day 0) and twice-weekly thereafter until study completion on day 14.

#### Assessment of mechanical hyperalgesia

Baseline paw pressure thresholds (PPTs) for each of the ipsilateral and contralateral hindpaws of FCA- and sham-rats were measured using the Randall-Selitto apparatus (Ugo Basile, Italy) as previously described (Randall and Selitto, 1957). Briefly, a noxious mechanical stimulus of increasing force was applied to the medial portion of the hindpaw until a withdrawal response was elicited. The maximum force applied was 250 g to prevent tissue damage. Baseline PPT values for each of the ipsilateral and contralateral hindpaws are the mean of three readings for the corresponding hindpaw, with a 5-min interval between consecutive measurements. The baseline PPTs were determined in both hindpaws prior to i.pl. FCA or saline injection (day 0) and thereafter twice-weekly until study completion at day 14.

#### Assessment of mechanical allodynia

Baseline paw withdrawal thresholds (PWTs) for each of the ipsilateral and contralateral hindpaws of CCI- or sham-rats were measured using calibrated von Frey filaments (Stoelting), as previously described (Ren, 1999; Muralidharan et al., 2013). Baseline PWTs were determined in both hindpaws prior to CCI- or sham-surgery and twice-weekly thereafter until study completion at day 14 post-surgery.

#### Assessment of burrowing behavior

Burrowing behavior is known to be altered by factors such as anxiety and distress (Jirkof et al., 2010). Hence, there were no humans present in the room during the burrowing experiment. The gravel used in the study was washed and dried prior to initiation of experiments in each cohort. For example, prior to initiation of burrowing experiments in Cohort 1, the gravel to be used in the study was washed and dried. The same set of gravel was used for on all experimental days until study completion at day 13/14. The gravel was not washed between different experimental days of Cohort 1. On completion of studies for Cohort 1 (i.e., on day 13/14), the gravel was washed and dried, and then used for studies in Cohort 2 in a similar fashion.

The burrowing apparatus comprised a normal rat cage (49.8 (L) × 38 (W) × 21.5 (H) cm) containing a hollow plastic burrowing tube (32 (L) × 10 (D) cm) sealed at one end and open at the other. The burrowing tube was filled with 2 kg of gravel (smooth; 3–5 mm diameter) and the open entrance was raised approximately 6 cm above the floor in the cage.

The burrowing assay was performed as described previously (Deacon, 2006; Andrews et al., 2012), with slight modifications. Briefly, all animals were habituated and trained to the burrowing conditions for 5 days prior to measurement of their baseline burrowing behavior. The training phase comprised social facilitation (Days −5 and −4) and individual training (Days −3, −2, and −1). All experiments were conducted toward the end of the light cycle (15:30 h). On the first day of social facilitation (Day −5), rats were placed in pairs into the testing cage with a burrowing tube filled with 2 kg of gravel for a period of 1 h. After 1 h, the amount of gravel displaced from the tube was weighed. At the end of experimentation, all rats were returned to their home cages. The same procedure was repeated on the second day of social facilitation (Day −4). If any rat pairs did not burrow at all on Day −5, then one rat from this pair was swapped with a rat from a burrowing pair (i.e., a rat that burrowed on Day −5) for social facilitation on Day −4.

On each day of individual training (Days −3, −2, and −1), rats were placed individually in a test cage containing a burrowing tube filled with 2 kg of gravel. The amount of gravel displaced from the tube after 1 h was weighed. At the end of 3 days of individual training, rats that burrowed ≤ 450 g of gravel were excluded from further participation in the study. Baseline burrowing data were collected from the remaining rats that burrowed ≥450 g on at least 2 out of 3 days of individual training. The baseline burrowing behavior was assessed prior to unilateral CCI/sham surgery or unilateral i.pl. FCA/saline injections, and then twice-weekly thereafter until study completion at day 14 post pain model induction.

### Statistical analyses

Statistical analyses were performed using repeated measures two-way analysis of variance (ANOVA) followed by the Bonferroni test to assess between-group differences in pain hypersensitivity behaviors (mechanical hyperalgesia and mechanical allodynia in FCA- and CCI-rats respectively), hindpaw volume (FCA-rats only), burrowing behavior and body weight data. Pearson's correlation analysis was used to compare burrowing performance with mechanical allodynia and mechanical hyperalgesia in individual rats. The *F*-values together with their associated degrees of freedom (treatment, time, interaction and residual) are reported as F _(df of treatment, time, interaction/residual)_. Statistical analyses were performed using GraphPad Prism™ v6.04 (GraphPad software Inc., San Diego, CA, USA) and the statistical significance criterion was *P* ≤ 0.05.

## Results

**Figure 1:**
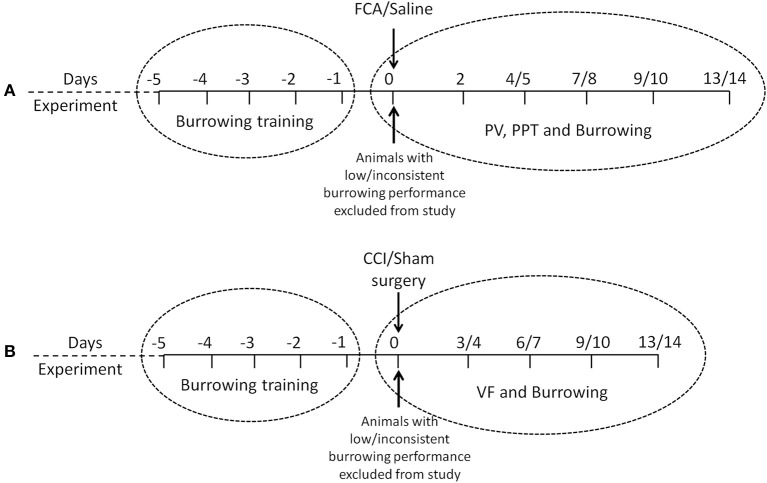
**Experimental design and chronological order of experimental procedures and/or behavioral testing used in the rat models of (A) FCA-induced inflammatory pain and (B) CCI of the sciatic nerve model of neuropathic pain**. PV, paw volume; PPT, Paw Pressure Threshold; VF, von Frey.

The timelines for assessments of burrowing behavior, mechanical hyperalgesia, hindpaw edema and/or mechanical allodynia in the FCA- and CCI-rat models used in the present study, are summarized in Figures 1A,B, respectively. The number of rats used per cohort and the number of animals excluded because they did not meet the burrowing criterion, are shown in Table 1. The general animal health (body weights), pain hypersensitivity behaviors (mechanical allodynia and mechanical hyperalgesia for CCI- and FCA-rats respectively), hindpaw volumes (FCA-rats only) and burrowing data from all rat cohorts are described in the following sections.

**Table 1:** **Details of cohort sizes used for each rodent pain model described in the present study**.

**Cohort**	**Number of animals used**	Number of animals excluded from the study^*^
**RAT MODEL OF CHRONIC INFLAMMATORY PAIN**		
1	10	3
2	10	5
3	10	3
**RAT MODEL OF NEUROPATHIC PAIN**		
1	16	3
2	10	4
3	10	0
4	10	2

* *Number of animals excluded because they did not meet the burrowing criterion*.

### General animal health

**Figure 2:**
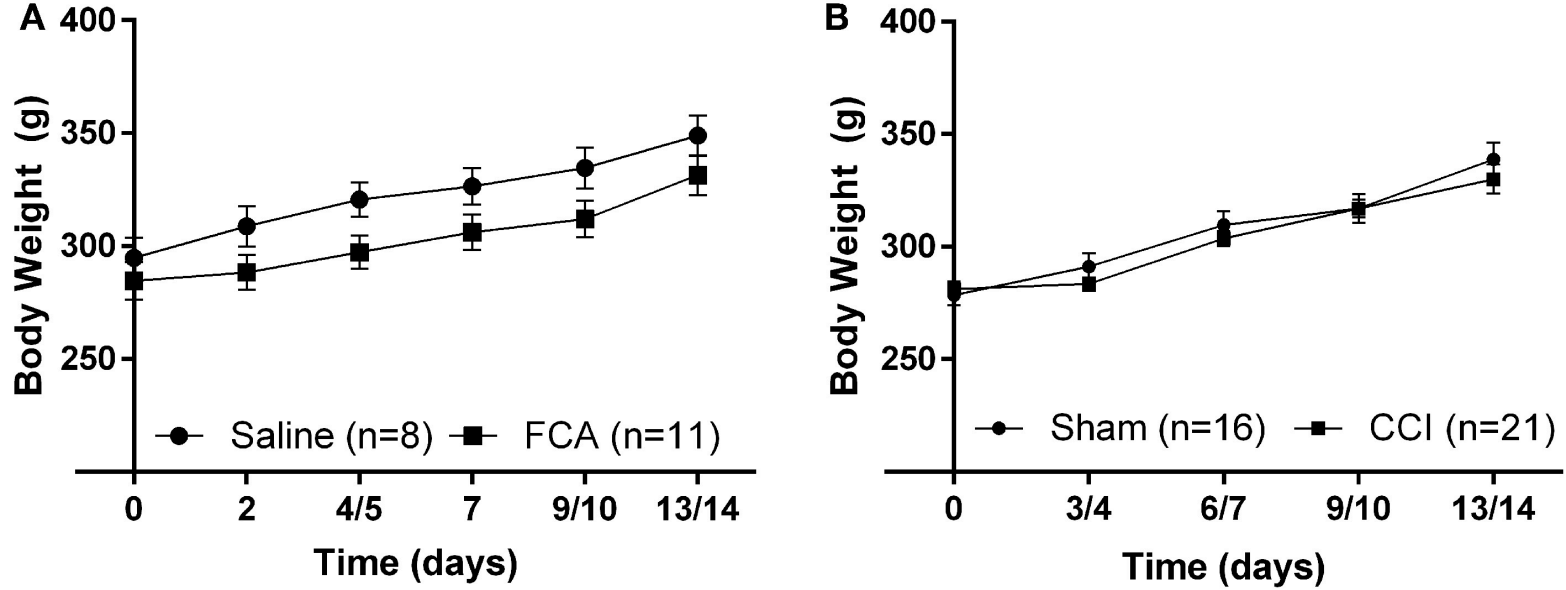
**Cumulative mean (±SEM) body weight vs. time curves for (A) Cohorts 1–3 rats administered a unilateral i.pl. injection of FCA (*n* = 11) or saline (*n* = 8), and (B) Cohort 1–4 rats that underwent CCI (*n* = 21) or sham (*n* = 16) surgery**. For FCA- and CCI-rats, there were no significant (*P* > 0.05) differences in the mean (±SEM) body weights throughout the experimental period when compared with that of their respective sham-control rats.

The mean (± SEM) body weights of FCA-rats from Cohorts 1–3 (Figure 2A) did not differ (*P* > 0.05) throughout the experimental period from the corresponding groups of sham-rats administered a unilateral i.pl. injection of saline.

For CCI- and sham-rats, there were no between-group differences (*P* > 0.05) in mean (± SEM) body weights during the study period (Figure 2B).

### Development of ipsilateral hindpaw edema in FCA-rats

**Figure 3:**
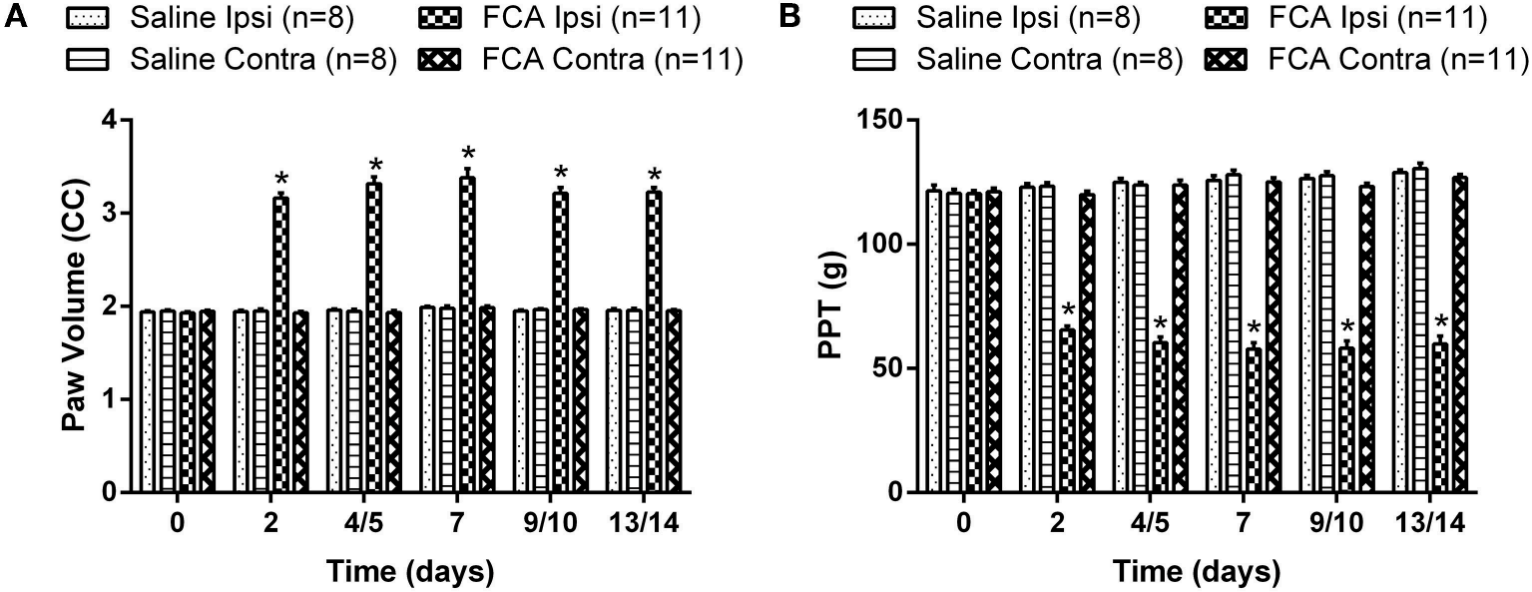
**The cumulative (A) mean (±SEM) PV and (B) mean (±SEM) PPT vs. time curves for Cohorts 1–3 rats administered a unilateral ip.l. injection of FCA (*n* = 11), relative to that of rats that received a unilateral ip.l. injection of saline (*n* = 8)**. Between days 2 and 14 post-ip.l. injection, there was significant (*P* ≤ 0.05) temporal development of edema and mechanical hyperalgesia in the ipsilateral hindpaws of FCA-rats *c.f*. the corresponding values for the ipsilateral hindpaws of sham-rats. Ipsi, Ipsilateral hindpaw; Contra, Contralateral hindpaw. ^*^P ≤ 0.05 (Two-way ANOVA, *post-hoc:* Bonferroni) relative to saline-injected sham-rats.

The mean (±SEM) ipsilateral PV vs. time curve data (Figure 3A) show that it was significantly increased between days 2 and 14 post i.pl. FCA injection [*F*_(3, 5, 15/170)_ = 582.8, 63.3, 64.4; *P* ≤ 0.05] compared with the corresponding data for rats administered unilateral i.pl. injections of saline or the contralateral hindpaw of FCA-rats for the 14-day period following i.pl. injection (Figure 3A).

### Development of mechanical hyperalgesia in the ipsilateral hindpaws of FCA-rats

The mean (±SEM) PPT vs. time curve data for FCA- and sham-rats in Cohorts 1–3 are shown cohort by cohort, in Supplementary Figures 1A–C, respectively. The mean (±SEM) PPT data (Figure 3B) for FCA-rats show that mechanical hyperalgesia was fully developed in the ipsilateral hindpaws from day 2 until study completion on day 14 [*F*_(3, 5, 15/170)_ = 586.6, 31.6, 64.7; *P* ≤ 0.05]. By contrast, mechanical hyperalgesia did not develop in the ipsilateral hindpaws of sham-rats or the contralateral hindpaws of FCA-rats (Figure 3B).

### Development of mechanical allodynia in the ipsilateral hindpaws of CCI-rats

**Figure 4:**
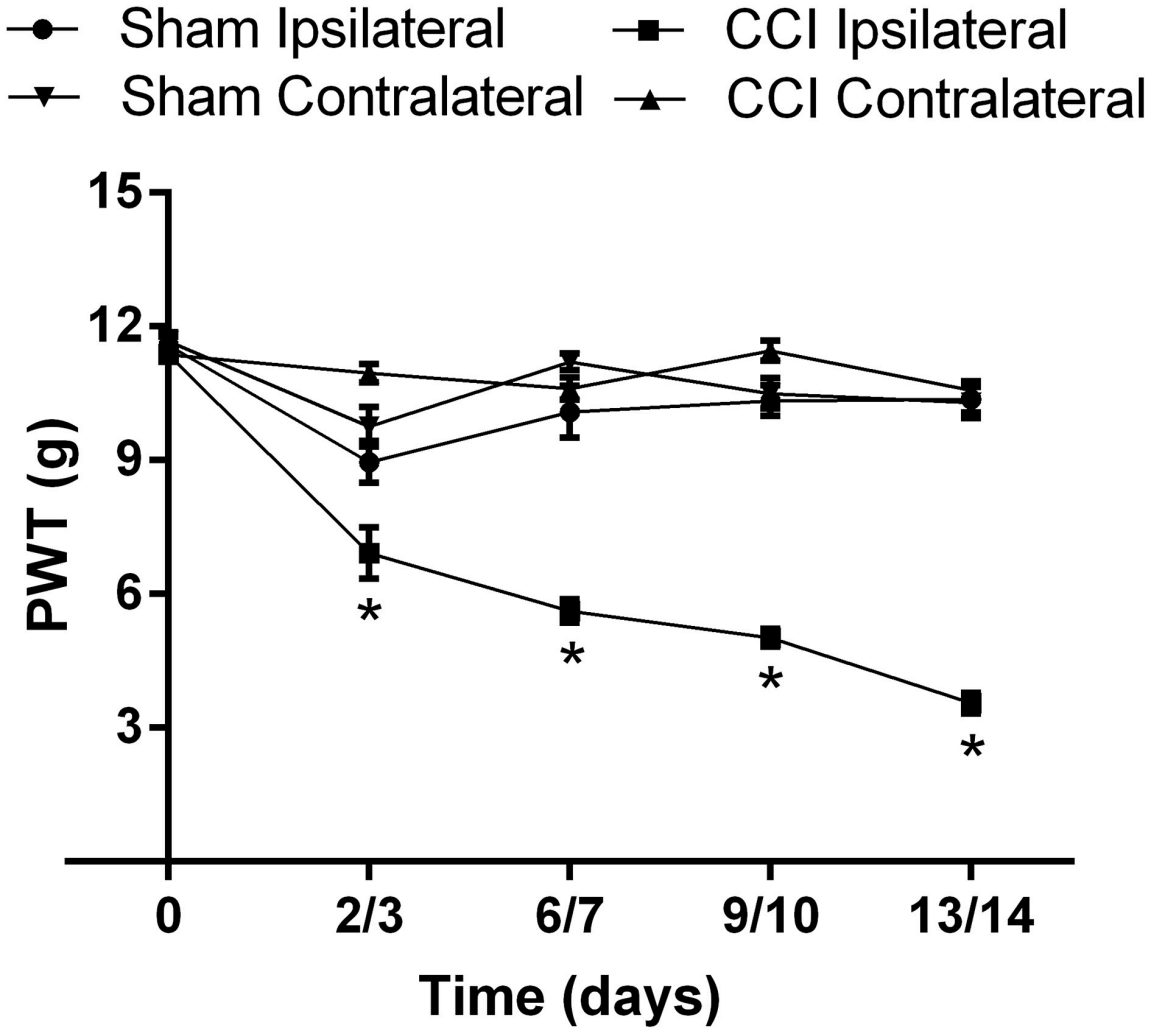
**The cumulative mean (±SEM) PWT vs. time curves for Cohorts 1–4 CCI- (*n* = 21) and sham (*n* = 16)-control rats**. For CCI-rats, there was a significant (*P* > 0.05) temporal reduction in the mean (±SEM) PWTs of the ipsilateral hindpaw between days 2 and 14 post-CCI surgery, relative to that of the mean (±SEM) PWT values of the ipsilateral hindpaw of sham-control rats or the contralateral hindpaw of CCI-rats. ^*^P ≤ 0.05 (Two-way ANOVA, *post-hoc:* Bonferroni) relative to sham-group rats.

The mean (±SEM) PWT vs. time curve data for CCI- and sham-rats in Cohorts 1–4 are shown cohort by cohort, in Supplementary Figures 2A–D, respectively. For the mean (±SEM) PWT data, there was significant [*F*_(3, 4, 12/280)_ = 165.6, 55.2, 24.7; *P* ≤ 0.05] temporal development of mechanical allodynia in the ipsilateral hindpaws of CCI-rats (Figure 4), between days 3 and 14 post-surgery, in contrast to the lack of change in the corresponding mean (±SEM) PWTs for the ipsilateral hindpaws of sham-control rats (Figure 4).

### Temporal changes in burrowing behavior in rodent models of chronic inflammatory pain and neuropathic pain

**Figure 5:**
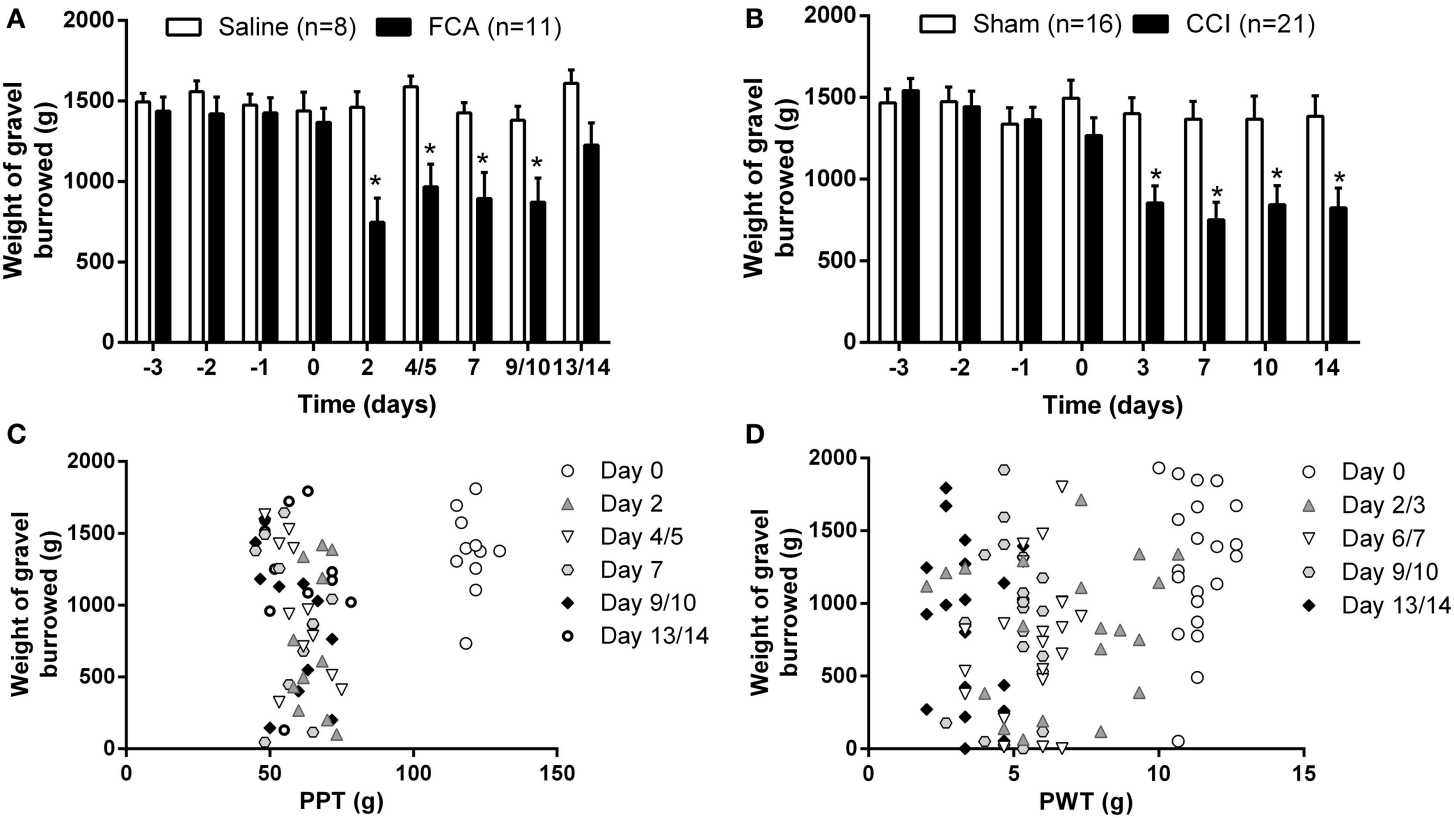
**Cumulative mean (±SEM) weight of gravel burrowed vs. time curves for (A) Cohorts 1–3 rats administered a unilateral ip.l. injection of FCA (*n* = 11) or saline (*n* = 8), and (B) Cohorts 1–4 rats that underwent CCI (*n* = 21) or sham (*n* = 16) surgery**. **(C,D)** Correlation between the degree of burrowing performance and mechanical hyperalgesia or mechanical allodynia for individual **(C)** FCA- and **(D)** CCI-rats tested. At days 2–10 following a unilateral i.pl. injection of FCA, there was a significant (*P* ≤ 0.05) temporal reduction in burrowing behavior compared with insignificant changes in the burrowing behavior of sham-rats. For CCI-rats, there was a significant (*P* ≤ 0.05) reduction in the mean (±SEM) weight of gravel burrowed between days 2 and 14 post-CCI surgery *c.f*. the corresponding values for the sham-control rats. Significant correlation between burrowing performance and mechanical hyperalgesia was observed only on day 4/5 in FCA-rats, in contrast to the lack of significant correlation between burrowing behavior and the extent of mechanical allodynia in the ipsilateral hindpaws of CCI-rats. ^*^P ≤ 0.05 (Two-way ANOVA, *post-hoc:* Bonferroni) relative to that observed in the corresponding groups of sham-control rats.

For rats in Cohorts 1–3 that received a unilateral i.pl. injection of FCA, there was a significant temporal decrease in the cumulative mean (±SEM) weight of gravel burrowed [*F*_(1, 8, 8/136)_ = 9.15, 6.03, 4.36; *P* ≤ 0.05] between days 2 and 10 in contrast to the insignificant change in the weight of gavel burrowed by sham-rats across the 14-day experimental period (Figure 5A). Likewise, between days 3 and 14 post-surgery, there was a significant [*F*_(1, 7, 7/245)_ = 7.9, 10.4, 6.8; *P* ≤ 0.05] temporal decrease in the burrowing behavior of rats with a unilateral CCI of the sciatic nerve relative to that of sham-control rats (Figure 5B). However, there was significant between-cohort variability in the burrowing behavior of both FCA- and CCI-rats relative to that of their respective sham-controls (Supplementary Figures 3A–C, 4A–D, respectively; see the respective supplementary figure legends for individual cohort statistical analyses). Specifically, comparison of the burrowing behavior of FCA-rats between the different cohorts showed significant [*F*_(2, 5, 10/40_ = 3.2, 9.3, 2.2; *P* ≤ 0.05] between-cohort variability in burrowing behavior on days 2 and 4/5 post-i.pl. FCA injection (Supplementary Figure 5A). Likewise, For CCI-rats, there was significant [*F*_(3, 4, 12/68_ = 2.4, 7.3, 2.3; *P* ≤ 0.05] between cohort variability in burrowing behavior on day 14 post-CCI surgery (Supplementary Figure 5B). In contrast, there were no between-cohort differences in the burrowing behavior of saline-injected rats [*F*_(2, 5, 10/25_ = 2.3, 1.4, 0.6; Supplementary Figure 5C] or sham surgery animals [*F*_(3, 4, 12/48_ = 1.4, 0.8, 1.2; Supplementary Figure 5D]. Although different cohorts had different experimenters, the between-experimenter differences are mitigated as testers are not present in the room during the burrowing sessions.

Importantly, there was a significant correlation (*P* ≤ 0.05) between burrowing performance and the extent of development of mechanical hyperalgesia in the ipsilateral hindpaws on day 4/5 post-i.pl. FCA injection in FCA-rats (Figure 5C). By contrast, for CCI-rats, burrowing performance was not correlated significantly (*P* > 0.05) with von Frey PWT values in the hindpaws throughout the experimental period (Figure 5D).

## Discussion

Our present findings show a significant decrease in ethologically-relevant burrowing behavior as well as development of mechanical hyperalgesia and mechanical allodynia in the ipsilateral hindpaws of FCA-rats and CCI-rats, respectively. Although the sensitivity of the burrowing test was comparable with that of noxious mechanical stimulus-evoked pain behavior in the same animals, inter-cohort variability was much greater for burrowing behavior (Supplementary Figures 3A–C, 4A–D) compared with mechanical hypersensitivity in the hindpaws (Supplementary Figures 1A–C,2A–D). In practical terms, this means that for experiments that utilize burrowing behavior as the primary pain behavioral endpoint in rodent models of inflammatory and neuropathic pain, larger group sizes will be needed to ensure they are adequately powered compared with similar experiments where mechanical allodynia or hyperalgesia in the ipsilateral hindpaws, is used as the primary endpoint.

Our afore-mentioned findings are aligned with previous work by others that showed reduced burrowing behavior in rats with FCA-induced inflammatory pain of the hindpaw (Andrews et al., 2012; Gould et al., 2016). The utility of assessing changes in burrowing behavior as a measure of spontaneous pain in rat models of peripheral neuropathy (Huang et al., 2013; Lau et al., 2013; Percie du Sert and Rice, 2014) and post-surgical pain (Jirkof et al., 2010) has also been demonstrated. Importantly, these studies collectively show considerable inter-rat variability in burrowing behavior (Andrews et al., 2012; Rutten et al., 2014a,b) in a manner similar to that reported herein.

The recognition and/or assessment of pain behavior in laboratory rodents have historically been done using reflex-withdrawal based behavioral paradigms (Mogil et al., 2010; Percie du Sert and Rice, 2014). Apart from the potential for methodological problems with these subjective stimuli-evoked pain behavioral outcomes (Berge, 2014), they fail to measure cognitive appraisal or the global impact of pain (Andrews et al., 2012). Hence, evaluation of ethologically relevant rodent behavioral endpoints, that may capture elements of ongoing pain and/or disability, has gained considerable momentum (Percie du Sert and Rice, 2014; Tappe-Theodor and Kuner, 2014). This is being driven by the on-going large translational gap between promising analgesic efficacy data generated in rodent chronic pain models and the ability of promising compounds so identified, to produce analgesia in early phase clinical trials conducted in patients with chronic pain of various etiologies (Percie du Sert and Rice, 2014; Tappe-Theodor and Kuner, 2014).

In the past 5 years, multiple measurable surrogate pain behavioral endpoints have been shown to be altered in rodent models of pathological pain. These include voluntary wheel running, burrowing, place preference and facial expression analysis (King et al., 2009; Langford et al., 2010; Andrews et al., 2012; Cobos et al., 2012; Rutten et al., 2014a). However, as burrowing is thought to be indicative of global well-being in rodents, and pain affects both well-being and contact-induced activity, altered burrowing behavior in particular, appears to have good face validity as a surrogate pain behavioral end-point (Andrews et al., 2012).

In the present study and subsequent work in our laboratory, it is apparent that the normal range for baseline burrowing of gravel in young adult male Sprague Dawley rats is in the range 1400–1700 g over a 1 h burrowing period. Whilst there were significant reductions (30–50%) in burrowing performance following a unilateral i.pl. injection of FCA or unilateral CCI of the sciatic nerve, marked inter-cohort variability was observed in both experimental groups. It is also possible that the observed reduction in burrowing performance may be associated with the presence of persistent pain in the hindpaws, resulting in reduced locomotor activity. However, absence of a significant correlation between the extent of burrowing performance and hindpaw hypersensitivity in individual CCI-rats and FCA-rats, in agreement with previous studies (Andrews et al., 2011), suggests that the impaired burrowing observed was not due to avoidance of pain in the hindpaws or to pain caused by the burrowing process itself. Instead, it may result from chronic pain that affects the motivation to burrow. This notion is supported by previous work by others that showed reduced burrowing behavior in rats with stavudine-induced neuropathy (Huang et al., 2013) and in mice with post-laparotomy pain (Jirkof et al., 2010), a model where the hind limbs are not directly affected. Although other indices of burrowing performance, *viz*. latency to onset of burrowing or duration of burrowing could have been measured in the present study, previous research by others showed negligible impact on such indices in rodent models of pathological pain (Jirkof et al., 2010).

In contrast to burrowing, mechanical hyperalgesia and mechanical allodynia were robust pain behavioral measures that showed insignificant inter-cohort variability. Our present data demonstrating reproducibility of these noxious mechanical stimulus-evoked pain behavioral assays in the ipsilateral hindpaws recapitulate historical data from our laboratory (Smith et al., 2002, 2013) as well a large body of previous work by others (Berge, 2011). Importantly, neither of these mechanical hypersensitivity behaviors developed in the contralateral hindpaws of FCA- or CCI-rats, or in either hindpaw of sham-rats.

Most of the evoked pain behavioral assays require that animals be restrained in the testing environments, thereby potentially resulting in false positives/negatives due to stress-induced analgesia, especially if the animals are not adequately habituated to the testing environment prior to experimentation (Mogil, 2009). However, the incidence of false positives/negatives can be minimized by evaluation of innate burrowing behaviors in an environment to which animals have been acclimatized. Importantly, as burrowing is assessed in a room with the experimenter outside, between-experimenter differences as well as subjective experimenter bias is avoided. The burrowing assay may also have potential for identification of drug doses that evoke pain relief but without potentially confounding sedative side-effects that would otherwise impair motor function (Andrews et al., 2012; Rutten et al., 2014a).

In conclusion, the construct validity of burrowing as a surrogate measure of spontaneous pain in rodent models of chronic inflammatory and neuropathic pain appears to be good. Importantly, it is a paradigm that is ethologically relevant to rodents and a behavioral assay that is not confounded by experimenter bias. Due to the considerable inter-animal variability observed in burrowing behavior, larger animal group sizes will be needed to ensure that studies are adequately powered. The higher costs associated with increasing group sizes to adequately power burrowing as a pain behavioral endpoint is well-justified if it improves translation of promising preclinical data into positive proof-of-concept clinical trial outcomes in the novel pain therapeutics field.

## Author contributions

MS and AM designed the studies described herein. AM and AK performed the burrowing, hindpaw volume and mechanical hyperalgesia experiments in the FCA-rat model of inflammatory pain. JL and LM performed burrowing experiments in the CCI-rat model of neuropathic pain. MJ performed burrowing experiments in some cohorts of FCA-rats and CCI-rats. JN and LC contributed substantial knowledge and insights that were helpful to the design of the experiments, pertinent comments on the data generated and they critiqued manuscript drafts. AM and MS wrote the manuscript.

## Funding

AM, AK and this project were supported financially by funds from an Australian Research Council (ARC) Large Linkage grant (grant # LP120200623) in collaboration with Boehringer Ingelheim Pharma GmbH & Co. JN and LC are paid employees of Boehringer Ingelheim Pharma GmbH & Co.

### Conflict of interest statement

The authors declare that the research was conducted in the absence of any commercial or financial relationships that could be construed as a potential conflict of interest.

## Supplementary material

Supplementary Figure 1 **The mean (±SEM) PPT vs. time curves for Cohorts (A) one, (B) two, and (C) three rats administered a unilateral ip.l. injection of FCA (*n* = 3–4 per cohort) or saline (*n* = 2–3 per cohort)**. For FCA-rats, there was insignificant between-cohort variability (*P* > 0.05) in the temporal development of mechanical hyperalgesia in the ipsilateral hindpaws. Importantly, mechanical hyperalgesia did not develop in the ipsilateral hindpaws of the corresponding groups of sham-rats.

Supplementary Figure 2 **The mean (±SEM) PWT vs. time curves for Cohorts (A) one, (B) two, (C), three and (D) four rats that underwent CCI- (*n* = 3–9 per cohort) or sham (*n* = 3–5 per cohort)-surgery**. For CCI-rats, there was insignificant between-cohort variability (*P* > 0.05) in the temporal development of mechanical allodynia in the ipsilateral hindpaws. Mechanical allodynia did not develop in the ipsilateral hindpaws of the corresponding groups of sham-rats.

Supplementary Figure 3 **The mean (±SEM) weight of gravel burrowed vs. time curves for FCA (*n* = 3–4 per cohort) and saline-rats (*n* = 2–3 per cohort) in Cohorts (A) one [*F*_(1, 8, 8/40)_ = 13.4, 7.01, 7.9], (B) two [*F*_(1, 8, 8/24)_ = 0.9, 1.5, 1.1], and (C) three [*F*_(1, 8, 8/40)_ = 5.7, 1.7, 0.5], show considerable between-cohort variability in burrowing behavior**.

Supplementary Figure 4 **The mean (±SEM) weight of gravel burrowed vs. time curves for CCI- (*n* = 3–9 per cohort) and sham-rats (*n* = 3–5 per cohort) in Cohorts (A) one [*F*_(1, 7, 7/77)_ = 1.5, 6.8, 3.7], (B) two [*F*_(1, 7, 7/28)_ = 5.7, 5.2, 1.5], (C) three [*F*_(1, 7, 7/56)_ = 2.2, 1.2, 2.4], and (D) four [*F*_(1, 7, 7/42)_ = 1.9, 2.6, 1.8], show considerable between-cohort variability in burrowing behavior**.

Supplementary Figure 5 **The mean (±SEM) weight of gravel burrowed vs. time curves for various cohorts of (A) FCA- (*n* = 3–4 per cohort), (B) CCI- (*n* = 3–9 per cohort), (C) saline- (*n* = 2–3 per cohort), and (D) sham surgery-group (*n* = 3–5 per cohort) rats**. There was significant between-cohort variability in the burrowing behavior of FCA- (day 2 and 4/5) and CCI-rats (day 14). By contrast and importantly, there was no between-cohort variability for saline-injected rats in the inflammatory pain model experiments, or for sham-rats in the CCI-model experiments.
