# Supplementary figures and images for: Comparison of Burrowing and Stimuli-Evoked Pain Behaviors as End-Points in Rat Models of Inflammatory Pain and Peripheral Neuropathic Pain
Source: Front Behav Neurosci. 2016 May 10;10:88. doi: 10.3389/fnbeh.2016.00088 (PMC4862327; doi:10.3389/fnbeh.2016.00088)

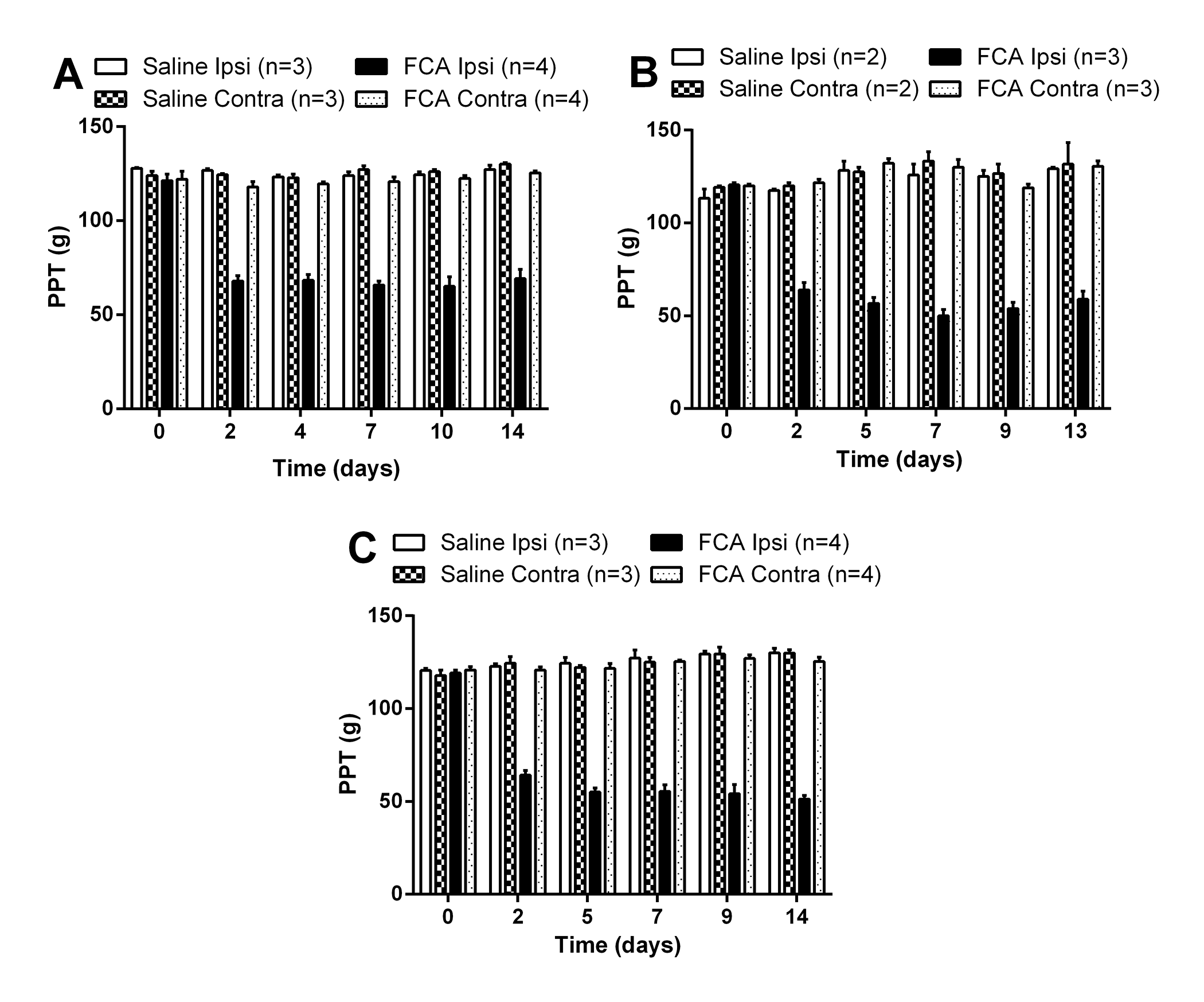

Supplement: Supplementary Figure 1 — The mean (±SEM) PPT vs. time curves for Cohorts (A) one, (B) two, and (C) three rats administered a unilateral ip.l. injection of FCA (n = 3–4 per cohort) or saline (n = 2–3 per cohort). For FCA-rats, there was insignificant between-cohort variability (P > 0.05) in the temporal development of mechanical hyperalgesia in the ipsilateral hindpaws. Importantly, mechanical hyperalgesia did not develop in the ipsilateral hindpaws of the corresponding groups of sham-rats. [file Image1.TIF]

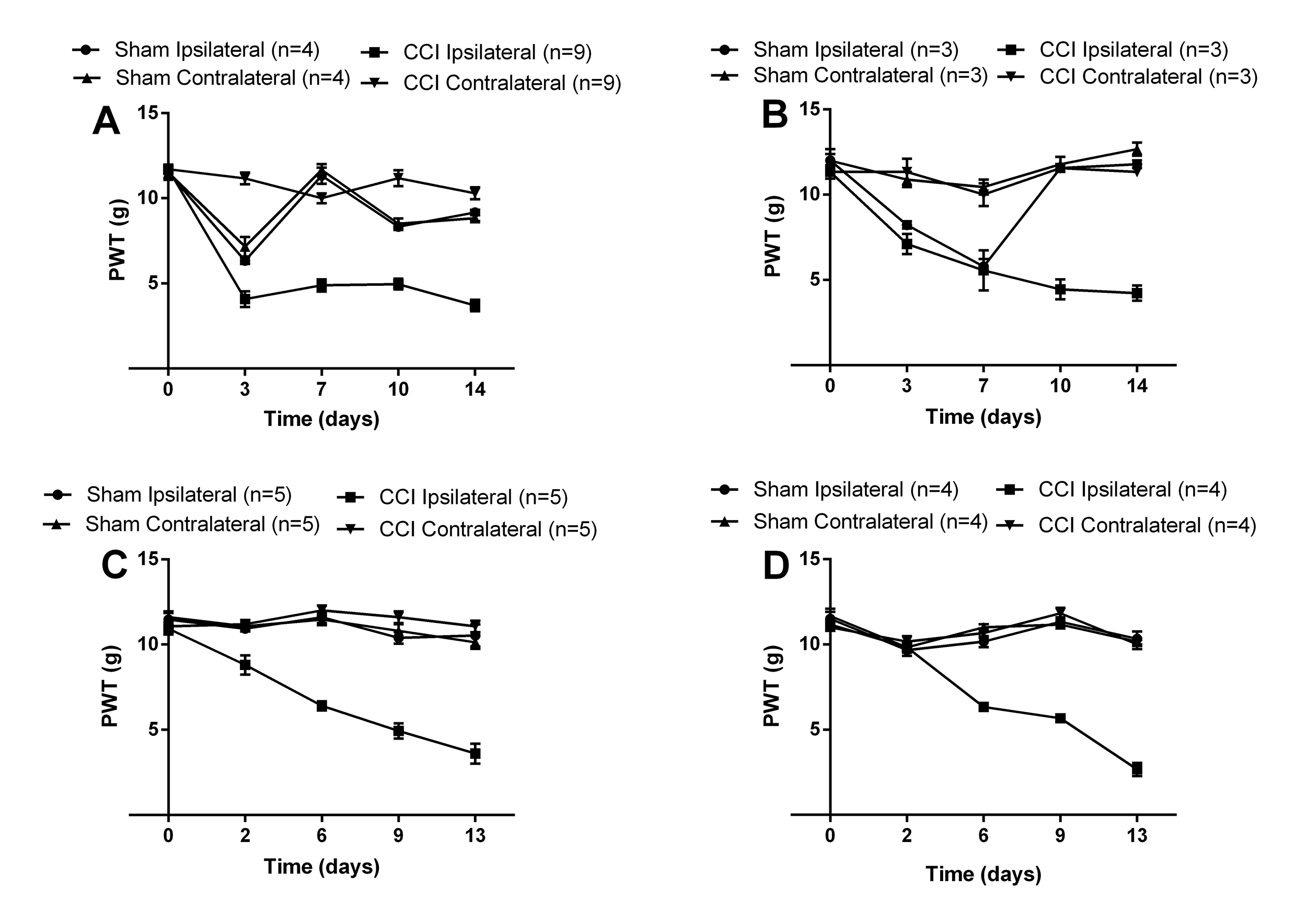

Supplement: Supplementary Figure 2 — The mean (±SEM) PWT vs. time curves for Cohorts (A) one, (B) two, (C), three and (D) four rats that underwent CCI- (n = 3–9 per cohort) or sham (n = 3–5 per cohort)-surgery. For CCI-rats, there was insignificant between-cohort variability (P > 0.05) in the temporal development of mechanical allodynia in the ipsilateral hindpaws. Mechanical allodynia did not develop in the ipsilateral hindpaws of the corresponding groups of sham-rats. [file Image2.TIF]

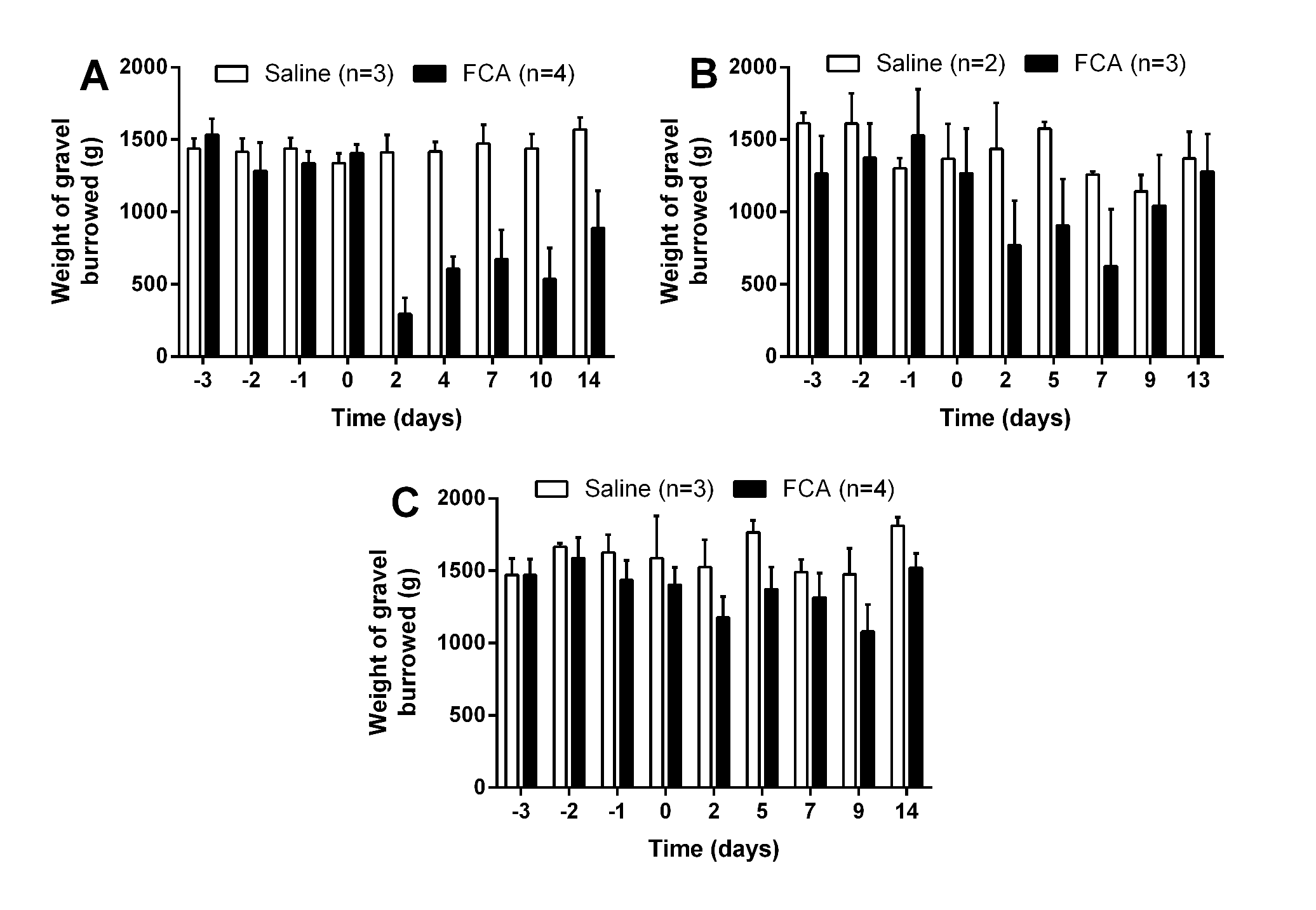

Supplement: Supplementary Figure 3 — The mean (±SEM) weight of gravel burrowed vs. time curves for FCA (n = 3–4 per cohort) and saline-rats (n = 2–3 per cohort) in Cohorts (A) one [F(1, 8, 8/40) = 13.4, 7.01, 7.9], (B) two [F(1, 8, 8/24) = 0.9, 1.5, 1.1], and (C) three [F(1, 8, 8/40) = 5.7, 1.7, 0.5], show considerable between-cohort variability in burrowing behavior. [file Image3.TIF]

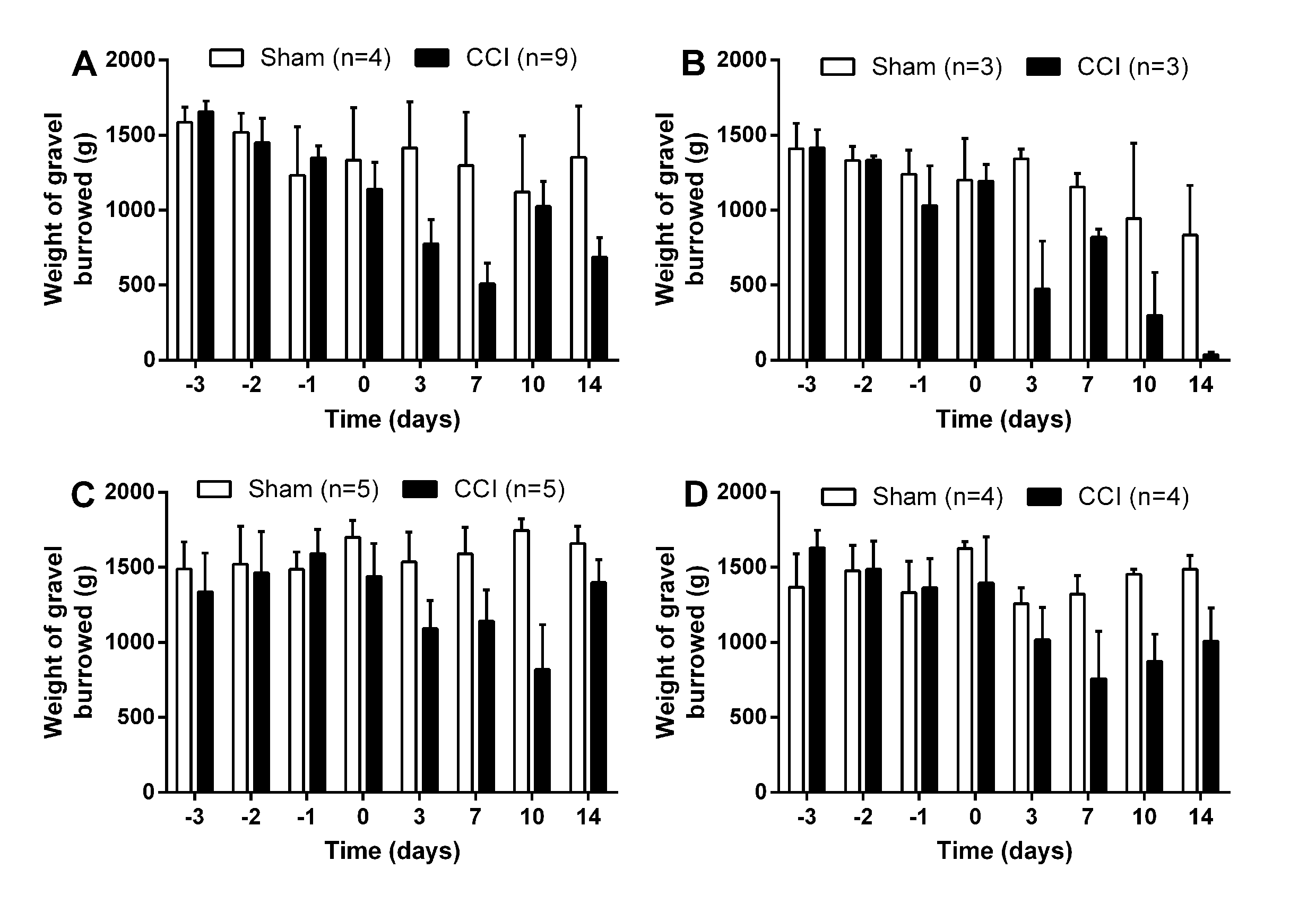

Supplement: Supplementary Figure 4 — The mean (±SEM) weight of gravel burrowed vs. time curves for CCI- (n = 3–9 per cohort) and sham-rats (n = 3–5 per cohort) in Cohorts (A) one [F(1, 7, 7/77) = 1.5, 6.8, 3.7], (B) two [F(1, 7, 7/28) = 5.7, 5.2, 1.5], (C) three [F(1, 7, 7/56) = 2.2, 1.2, 2.4], and (D) four [F(1, 7, 7/42) = 1.9, 2.6, 1.8], show considerable between-cohort variability in burrowing behavior. [file Image4.TIF]

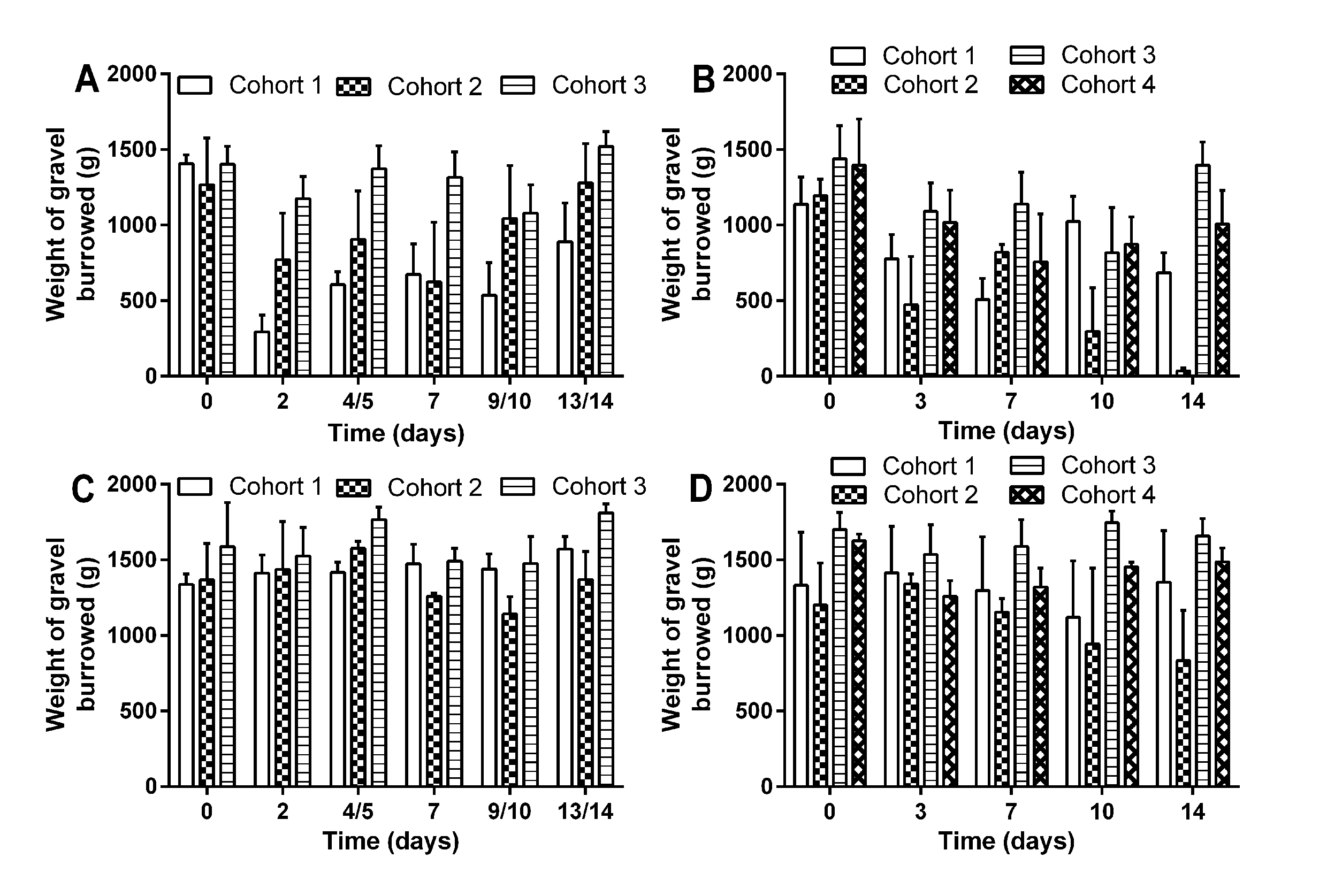

Supplement: Supplementary Figure 5 — The mean (±SEM) weight of gravel burrowed vs. time curves for various cohorts of (A) FCA- (n = 3–4 per cohort), (B) CCI- (n = 3–9 per cohort), (C) saline- (n = 2–3 per cohort), and (D) sham surgery-group (n = 3–5 per cohort) rats. There was significant between-cohort variability in the burrowing behavior of FCA- (day 2 and 4/5) and CCI-rats (day 14). By contrast and importantly, there was no between-cohort variability for saline-injected rats in the inflammatory pain model experiments, or for sham-rats in the CCI-model experiments. [file Image5.TIF]
